# Resveratrol Delivery by Albumin Nanoparticles Improved Neurological Function and Neuronal Damage in Transient Middle Cerebral Artery Occlusion Rats

**DOI:** 10.3389/fphar.2018.01403

**Published:** 2018-12-04

**Authors:** Huae Xu, Ye Hua, Jie Zhong, Xiaolin Li, Wei Xu, Yingyuan Cai, Yukang Mao, Xiaowei Lu

**Affiliations:** ^1^School of Pharmacy, Nanjing Medical University, Nanjing, China; ^2^Department of Neurology, The Affiliated Wuxi Second People’s Hospital of Nanjing Medical University, Wuxi, China; ^3^School of Foreign Languages, Nanjing University of Finance and Economics, Nanjing, China; ^4^Department of Geriatric Gastroenterology, The First Affiliated Hospital of Nanjing Medical University, Nanjing, China; ^5^Department of Geriatric Neurology, The First Affiliated Hospital of Nanjing Medical University, Nanjing, China; ^6^Department of Medical Image Science, Xuzhou Medical University, Xuzhou, China

**Keywords:** human serum albumin, resveratrol, nanoparticle, cerebral, ischemia-reperfusion

## Abstract

Human serum albumin (HSA) is an intrinsic protein and important carrier that transports endogenous as well as exogenous substances. It is demonstrated in this study that the regional accumulation of albumin in the ischemia-reperfusion (I/R) brain may lead in the application of HSA based nanoparticles in the study of cerebral I/R injury. Resveratrol (RES) is potential in the treatment of cerebral I/R injury but is restricted for its water insolubility and short half-life *in vivo*. In our study, RES loaded HSA nanoparticles (RES-HSA-NPs) were prepared to facilitate the application of RES in protection from cerebral I/R injury. RES-HSA-NPs demonstrated spherical shape, a diameter about 100 nm, a highest RES encapsulation efficiency of 60.9 ± 5.07%, and controlled release pattern with the maximum release ratio of 50.2 ± 4.91% [in pH = 5.0 phosphate buffered saline (PBS)] and 26. 2 ± 2.73% (in pH = 7.4 PBS), respectively, after 90 h incubation at 37°C. After intravenous injection into transient middle cerebral artery occlusion (tMCAO) rats, RES-HSA-NPs improved neurological score and decreased infarct volume at 24 h after tMCAO in a dose dependent manner. A single dose of 20 mg/kg RES-HSA-NPs via tail vein improved neurological outcomes and decreased infarct volume at 24 and 72 h in tMCAO rats. I/R increased oxidative stress (indicated by products of lipid peroxidation, MDA) and neuronal apoptosis (indicated by yellow-brown TUNEL-positive cells), RES-HSA-NPs significantly attenuated oxidative stress and neuronal apoptosis. These results demonstrated the potential of RES-HSA-NPs in the therapy of cerebral I/R injury.

## Introduction

The incidence of ischemic stroke (IS) increased quickly in older population with the aging process (Al AlShaikh et al., 2016). IS is the main cause for the loss of neurological functions and thus results in disability and death worldwide (Al AlShaikh et al., 2016). Recently, reperfusion therapy which is based on endovascular thrombectomy and intravenous thrombolysis has been greatly developed (Saver et al., 2015). However, the sudden recovery of blood flow results in cerebral ischemia-reperfusion (I/R) injury (Chamorro, 2018) and weakens the beneficial effect of reperfusion therapy.

The underlying mechanisms involved in cerebral I/R injury include excitotoxicity, overload of Ca^2+^, oxidative stress, inflammation, and apoptosis (Chamorro et al., 2016; Gupta and Ghosh, 2017; Chamorro, 2018). These mechanisms may have individual or combined action during I/R period. Oxidative stress, including excessive oxidation and insufficient elimination, has been proved to be a critical underlying molecular mechanism in the process of I/R injury (Chamorro et al., 2016; Nagy and Nardai, 2017; Yang J.-L. et al., 2018). It is supposed that the exogenous supply of antioxidants in conjunction with reperfusion therapy will attenuate oxidative stress and optimize the effect of reperfusion therapy.

Resveratrol (RES), a natural polyphenol, has been widely used as antioxidant and was reported to be able to attenuate oxidative stress, inflammation and apoptosis related to I/R injury (Ghosh et al., 2014; Bastianetto et al., 2015). However, due to poor water solubility, short half-life *in vivo* and low concentration in brain, its application was restricted (Nabavi et al., 2014; Bastianetto et al., 2015). It was found recently that drug delivery systems basing on encapsulation strategy could improve the therapeutic efficacy of drugs by the increase of stability *in vivo*, cellular/tissue uptake, and target accumulation (Patil and Deshpande, 2018; Zhang et al., 2018). Many encapsulation methods were used currently, such as liposomes, polymeric-based nanoparticles, hydrogels, and serum albumin (Madni et al., 2014; Parayath and Amiji, 2017; Sims et al., 2018; Yang K. et al., 2018). Among which, endogenous serum albumin has been reported as one of the most potential carriers for the delivery of insoluble drugs because of its non-toxicity, non-immunogenicity and great biocompatibility (Parayath and Amiji, 2017). Serum albumin was used as the macromolecular carrier in the delivery of drugs and led in the success of paclitaxel delivery (Chen et al., 2015; Parayath and Amiji, 2017).

It was reported in recent publication that macromolecules would passively target into the I/R region due to the opening blood–brain barrier (BBB) (He et al., 2018; Lv et al., 2018; Nair et al., 2018). In this paper, the localizing behavior of serum albumin into I/R-brain is demonstrated with the quantitation of plasma clearance and brain accumulation of Albumin-Evans-Blue complex in transient middle cerebral artery occlusion (tMCAO) rats. Human serum albumin (HSA) was also selected to load lipophilic RES. After HSA nanoparticles loaded with RES (RES-HSA-NPs) were characterized, the neuroprotective effect of RES-HSA-NPs on cerebral I/R injury was investigated in the tMCAO rat model.

## Materials and Methods

### Preparation of the tMCAO Rat Model

Adult, male Sprague–Dawley rats (weighed 250–300 g) were purchased from the National Rodent Laboratory Animal Resources (Shanghai Branch of China). These rats were housed at the relative humidity of 30–35% and the temperature of 19–25°C, with 12-h light/dark exposure and free access to chows and water. All animal procedures were approved and conducted in accordance with the Animal Ethics Review Committee regulations of Nanjing Medical University.

Transient focal cerebral ischemia models were prepared through the transient intraluminal filament middle cerebral artery occlusion (MCAO) method as reported previously (Lopez and Vemuganti, 2018). Briefly, rats were anesthetized with 2% isoflurane and maintained by 1.5% isoflurane in 70% nitrous oxide and 30% oxygen. The right common carotid and external carotid arteries of the rates were carefully exposed and then a 4-0 monofilament nylon suture (the distal 3 mm of filaments was coated with silicone resin/hardener mixture) was inserted into the internal carotid artery and advanced to the origin of MCA. The sham animals were prepared by only exposing the right common carotid and no suture was inserted. The middle cerebral artery was occluded for 2 h and the timepoint of filament withdrawn was defined as the beginning of reperfusion (reperfusion 0 h). The Laser-Doppler flowmetry (model DRT 4 2CH; Moor Instruments, Devon, United Kingdom) was applied to monitor the regional cerebra blood flow of ischemic area. The monitoring of cerebral blood flow (CBF) was conducted before, during, and immediately after the release of occlusion, respectively. The blood flow was kept lower than 30% of baseline after occlusion and reperfusion blood flow should be greater than 70% of baseline, which provided a more homogeneous group by excluding poor tMCAO rats and thus resulted in neurologic score narrowly ranging from 7 to 11. Systolic blood pressure was monitored non-invasively. Rectal temperature was maintained at 37.0°C ± 0.5°C on a homeothermic blanket. The pulse oximeter (Shanghai Berry Electronic Technology Co., Ltd, China) was used to monitor the tissue oxygen saturation from the paw. The rats were euthanized with pentobarbital (120 mg/kg) before the trans-cardial perfusion with 150 ml of heparinized saline (10 U/ml). The follow-up procedures of fixing brain tissues were conducted in solutions which were designed according to each specialized protocol, following perfusion with heparinized saline at a rate of 5 ml/min.

### Plasma Clearance and Accumulation of Albumin-Evans-Blue Complex in I/R Brain

In 1986, Matsumura firstly reported the accumulation behavior of albumin into tumors at the tissue level through quantitative analysis of plasma clearance and accumulation of Albumin-Evans-Blue Complex (Matsumura and Maeda, 1986). In published experiments about IS, Albumin-Evans-Blue Complex analysis was also used to visualize and measure the permeability of the BBB in rats (Reddy and Labhasetwar, 2009; Teng et al., 2017). In our experiment, modified quantitative analysis of the plasma clearance and accumulation of Albumin-Evans-Blue Complex into brain was used to indicate the localized behavior of Albumin into I/R brain. Briefly, Evans-blue dissolved at 0.2% in 0.9% saline solution was injected into the tail vein of the tMCAO rats at a dose of 10 mg/kg. At this dose level there was no free dye in the plasma; it was found mostly bound to albumin as revealed by Bio Gel P-10 gel filtration. The blood samples from the rat under ethyl ether anesthesia were obtained 0.2 ml at a time by cardiac puncture with a syringe fitted with a 27/32-gauge needle. The blood samples were immediately mixed with 2.8 ml of Isoton II (Coulter, Inc.) followed by centrifugation at 150 × *g* for 5 min. Then the concentration of Evans blue was determined spectrophotometrically at 620 nm (Nanodrop, Japan). Brains were removed and divided into two hemispheres (I/R-side and non-I/R-side). Hemispheres of I/R-side and non-I/R-side were, respectively, weighed and immersed into 3 ml of formamide followed by incubation at 60°C for 48 h to extract the dye. The concentration of the dye was similarly determined spectrophotometrically. The localization of HSA-Evans-Blue Complex into I/R brain was further visualized. Evans blue (1 mg) was mixed with HAS (8 mg) in 1 ml of saline in a test tube to prepare HSA*-Evans-Blue Complex* and then the complex was injected by tail vein tMCAO rat as a single dose. After 6 h of reperfusion, rat brain was harvested after transcardially perfused with heparinized saline. After that, digital photograph (Nikon D200, 7.1 megapixels) of the brain was taken.

### Formulation and Characterization of RES-HSA Nanoparticles

Resveratrol (≥99%) and HSA (lyophilized powder, ≥96%) were purchased from Sigma Chemical Co. (St. Louis, MO, United States). RES-HSA nanoparticles (RES-HSA-NPs) were synthesized with a modified but simple desolvation method (Guo et al., 2015). In detail, 6 mg RES was dissolved in DMSO to be 1 mg/ml and was mixed with 10 mg of HSA in 1 ml water under slighted stirring, forming hardened coacervates after stirring for 6 h under room temperature, and then was processed by cross-linking with 0.5% glutaraldehyde (100 μl). Afterward, the organic solvents were removed by dialyzing in water for 1 day, resulting in the RES-HSA-NPs. Empty-HSA nanoparticles (Empty-NPs) were prepared as above procedure by omitting RES. In determining the physical characteristic of nanoparticles, dynamic light scattering (DLS, Brookhaven BI-9000AT, United States) and transmission electron microscopy (TEM, JEM-100S, Japan) were used.

At the last step of preparation for RES-HSA-NPs, the organic solvents and free RES were removed by dialyzing in water for 1 day. The collected dialyzate was used to quantify the free RES by UV–Vis spectrometer at 306 nm according to a calibration curve. The drug concentration was calculated with a standard calibration curve. The encapsulation efficiency (%) = (the weight of total added RES-the weight of free RES)/the weight of total added RES × 100%.

A dynamic dialysis method was used to investigate the sustained release pattern of RES from the RES-HSA-NPs in PBS (pH = 5.0 and 7.4, respectively). The dialysis bag with a cutoff Mw of 8–12 kDa was used. Cumulative release (%) = the amount of released RES/total RES loaded in RES-HSA-NPs × 100%. The cumulative release pattern of RES from the RES-HSA-NPs was plotted in a function of time.

### Administration of RES-HSA-NPs via Tail Vein

The tail vein was isolated and cannulated with a PE-10 tube filled with PBS (Sigma-Aldrich, St. Louis). RES-HSA-NPs was dissolved in distilled water. The RES-HSA-NPs treatment group was injected with a single dose of 5, 10, 20, and 40 mg/kg RES-HSA-NPs via tail vein immediately (beginning of reperfusion) after 2-h occlusion. The vehicle treatment group was injected with the same volume of distilled water.

### Neurological Behavior Assessment

Neurological behavior was assessed by a blinded observer at 24 and 72 h after initiation of reperfusion according to a modified neurologic function scoring system (Li et al., 2001) as detailed in Table 1. The 14 points scoring system includes the following tasks: motor score (muscle status and abnormal movement), sensory score (visual, tactile, and proprioceptive), and reflex tests. One point accounted for the inability to perform the tasks correctly or the lack of a tested reflex. Severe lesion was suggested by the scores of 10–14, moderate 5–9, and mild 1–4. The higher the score, the more severe the lesion.

**Table 1:** Neurologic Function Scoring System.

Motor tests	Points
**Muscle status: hemiplegia**	
Raising the rat by the tail	
Flexion of forelimb	1
Flexion of hindlimb	1
Head moving more than 10° (vertical axis)	1
Placing the rat on the floor	
Inability to walk straight	1
Circling toward the paretic side	1
Falling down to the paretic side	1
Abnormal movements	
Immobility and staring	1
Tremor (wet-dog shakes)	1
Myodystony, irritability, seizures	1
Sensory tests	
Visual and tactile placing	1
Proprioceptive test (deep sensory)	1
Reflexes (blunt or sharp stimulation) absent of:	
Pinna reflex (a head shake when touching the auditory meatus)	1
Corneal reflex (an eye blink when touching the cornea with cotton)	1
Startle reflex (a motor response to a brief loud paper noise)	1
**Maximum points**	14

### Measurement of Infarct Volume

At the 24th and 72nd h of reperfusion, the rat brains (*n* = 5 per group) were rapidly removed after anesthetization with ketamine and decapitation. After being sliced into 2-mm-thick coronal sections, the brain sections were stained with standard 2% 2,3,5-triphenyltetrazolium chloride (TTC, Sigma-Aldrich) for 10 min at 37°C and then immersed in 10% formalin overnight. The infarct tissue remained unstained (white), whereas normal tissues were stained red. The infarcted territory was demarcated and then analyzed by Image J software (version 1.32, National Institutes of Health). Individual infarct volume of each section was calculated from the infarct area of each section multiplied by the thickness of the brain section. Total infarct volume of each brain was obtained from the sum of infarct volume of each section. Edema corrected infarct volume was calculated with the formula: corrected infarct volume (ml) = section thickness × (contralateral hemisphere area-no infarct area of ipsilateral hemisphere). The relative infarct volume was indicated as the percentage of total brain volume as follows: corrected infarct volume/total brain volume × %.

### Oxidative Stress Injury and Apoptosis

#### Determination of MDA

The lipid peroxidation products (malondialdehyde [MDA]) were determined by an LPO-586 kit (OxisResearch, Portland). Rats from three groups (*n* = 4 for each group) were anesthetized and humanely killed at 24 h after reperfusion and then transcardially perfused with cold PBS. Right cerebral cortexes were homogenized in 20 mmol/l pH 7.4 phosphate buffer and 0.5 mol/l butylated hydroxytoluene in acetonitrile. After that, the homogenates were centrifuged at 3,000 × *g* for 10 min at 4°C. DC protein assay (Bio-Rad) was used to determine the protein concentration. Same amounts of proteins in each sample were reacted with a chromogenic reagent at 45°C for 60 min and then centrifuged at 15,000 × *g* for 10 min at 4°C. The supernatants were investigated spectrophotometrically at 586 nm. According to the standard curve supplied by the kit, the level of MDA was calculated into picomoles per milligram protein.

#### Quantification of TUNEL-Labeled Cells

After 72 h reperfusion, the rats were anesthetized and transcardially perfused with 0.9% saline followed by 4% paraformaldehyde. The harvested brain samples (*n* = 5, each group) were fixed overnight in 4% paraformaldehyde and then sliced into paraffin-embedded coronal sections (4 μm). An apoptosis detection kit (Boster, Wuhan, China) was used to conduct TUNEL staining. Then the samples were coverslipped and examined by an image analysis system (Carl Zeiss, CA, United States).

### Statistical Analysis

All data were expressed as the mean ± SEM. Differences among groups were tested by one-way ANOVA. Comparisons between two groups were evaluated with Student’s *t*-test. A probability value of *P* < 0.05 was considered as statistically significant.

## Results

### Plasma Clearance and Accumulation in I/R Brain of Albumin-Evans-Blue Complex

**FIGURE 1:**
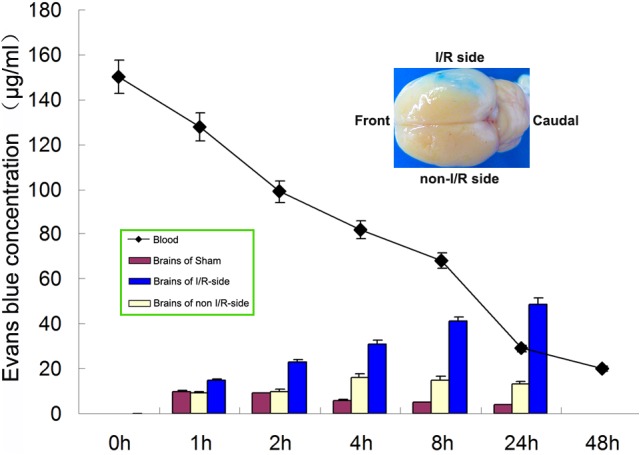
I/R Brain accumulation as revealed with Albumin-Evans-Blue Complex. Quantification of Evans blue at different times in plasma, brain of sham group, I/R-side hemisphere, and non-I/R-side hemisphere was illustrated, which shows a significant gradual increase in the concentration of I/R-side hemisphere but a slight increase of non-I/R-side. Plasma concentration progressively decreased, concentration in I/R-side became much higher than that of plasma 24 h after injection. The small picture on the right up showed a typical blue I/R-side compared with non-I/R, which revealed Evans-Blue-HSA Complex mainly localized into I/R-side.

The I/R-side hemisphere is blue compared with non-I/R-side. Quantification of Evans blue at different times in plasma, brain of sham group, I/R-side hemisphere, and non-I/R-side hemisphere was illustrated, which shows a significant gradual increase in the concentration of I/R-side hemisphere but a slight increase of non-I/R-side compared with Sham group. Plasma concentration progressively decreased, concentration in I/R-side became much higher than that of plasma 24 h after injection (Figure 1). HSA-Evans-Blue Complex mainly localized into the I/R-side hemisphere (the small picture on right up of Figure 1).

### Characterization of RES-HSA-NPs

**FIGURE 2:**
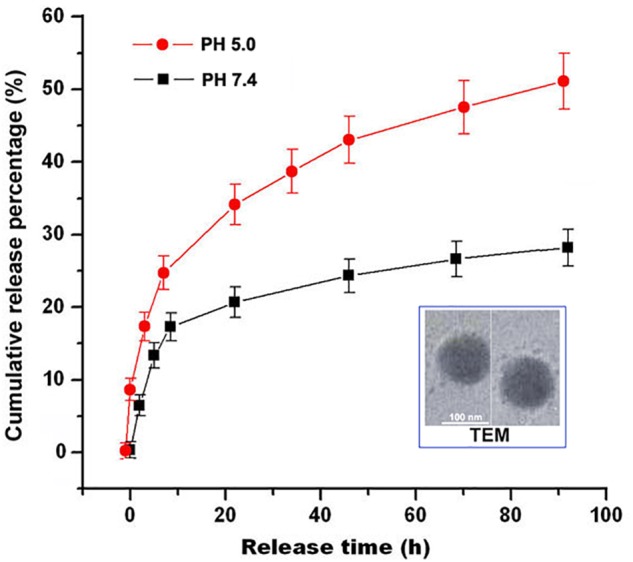
Characterization of RES-HSA-NPs. RES-HSA-NPs were almost spherical morphology demonstrated by transmit electron micrograph (TEM). Scale bar = 100 nm. RES release from RES-HSA-NPs in PBS (pH 5.0 and 7.4) presented as a sustained pattern by plotted as a function of time.

Measured by TEM, RES-HSA-NPs showed mostly spherical shape (Figure 2). The mean hydrodynamic diameter of RES-NPs as determined by DLS was about 100 nm. By changing the amount of total added RES, the obtained highest encapsulation efficiency of RES in RES-HSA-NPs was 60.9 ± 5.07%. RES-HSA-NPs exhibited a sustained release pattern of RES both in pH 5.0 and pH 7.4 PBS. After 90 h at 37°C, the highest release rates of RES were 50.2 ± 4.91 and 26.2 ± 2.73%, respectively. It has been reported that the blood pH is 7.4, while I/R brain is acidic (Yermolaieva et al., 2004). This release pattern provided benefit for using RES-HSA-NPs in I/R therapy.

### Physiological Data

There were no statistical differences between vehicle group and RES-HSA-NPs group in mean arterial blood pressure, heart rate, or glucose levels before and after ischemia (Table 2).

**Table 2:** Physiological data of preliminary five representative rats from vehicle group and RES-HSA-NPs group before and after ischemia.

	Before			After		
	Vehicle	NPs	*p*-value	Vehicle	NPs	*p*-value
MAP (mmHg)	179 ± 15	181 ± 12	> 0.05	191 ± 23	189 ± 25	> 0.05
Heart rate (beat/min)	297 ± 19	291 ± 21	> 0.05	337 ± 17	341 ± 23	> 0.05
Glucose (mmol/L)	3.9 ± 1.1	3.7 ± 1.3	> 0.05	3.1 ± 1.5	2.9 ± 1.7	> 0.05

*MBP, mean arterial pressure. NPs, RES-HSA-NPs.*

### Dose and Time Dependent Neuroprotection of RES-HSA-NPs From tMCAO

**FIGURE 3:**
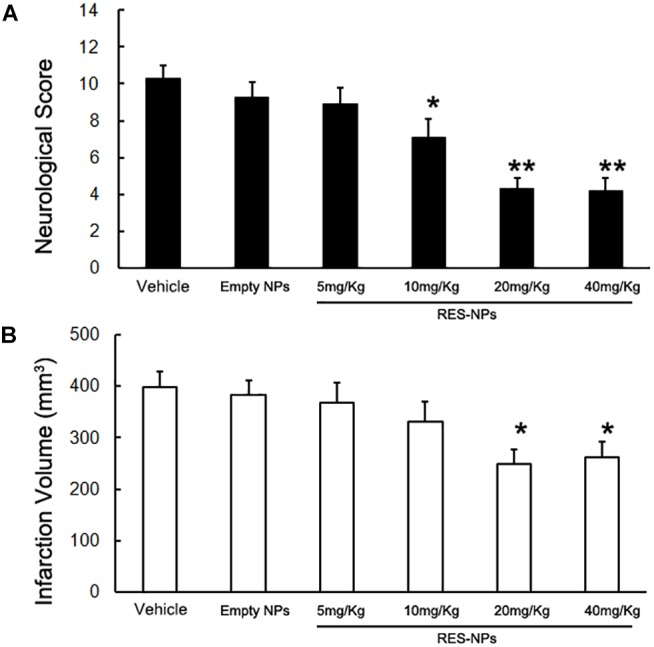
Reduction of neurological score and infarct volume by RES-HSA-NPs in rats following ischemia-reperfusion. At the time of reperfusion following 2 h of ischemia, rats received via tail vein either vehicle (control), Empty-HAS-NPs (Empty-NPs), or a suspension of different doses of RES-HSA-NPs (RES-NPs). Animals were euthanized 24 h after reperfusion. **(A)** Bar graph showing quantification of neurological scores from the vehicle, Empty-NPs, and RES-NPs treated rats. **(B)** Bar graph showing quantification of infarction volume from the PBS, Empty-NPs, and RES-NPs treated rats. Data are means ± SD. ^∗^*P* < 0.05 vs. PBS control, ^∗∗^*P* < 0.01 vs. vehicle (*n* = 5 per group).

**FIGURE 4:**
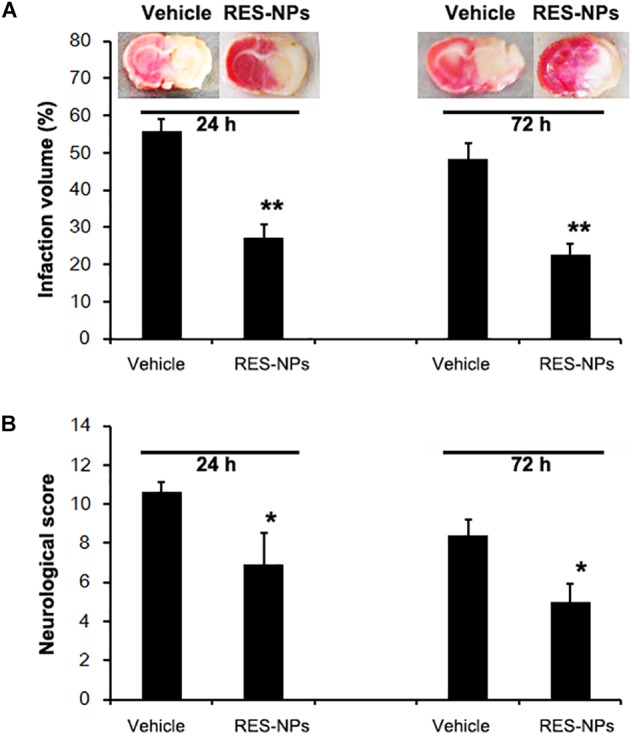
Reduction of neurological score and infarct volume by RES-HSA-NPs (RES-NPs) in rats suffering ischemia-reperfusion. At the onset of reperfusion following 2 h of ischemia, rats received via tail vein either vehicle (control) or a suspension of 20 mg/kg of RES-HSA-NPs. Animals were euthanized 24 and 72 h following reperfusion, respectively. **(A)** Representative coronal brain sections stained with TTC solution from vehicle or RES-NPs treated rats. Red colored regions in the TTC-stained sections indicate non-ischemic areas; pale-colored regions indicate ischemic portions. Bar graph showing quantification of infarction volume from the vehicle and RES-NPs treated rats. **(B)** Bar graph showing quantification of infarction volume from the vehicle and RES-NPs treated rats. Data are means ± SD. ^∗^*P* < 0.05 vs. PBS control, ^∗∗^*P* < 0.01 vs. vehicle (*n* = 5 per group).

RES-HSA-NPs treatment in incremental doses (mean RES doses: 5, 10, 20 and 40 mg/kg) resulted in a significant improvement of neurological score and decrease in infarct volume 24 h after tMCAO (Figures 3A,B). Maximal beneficial effects were observed at the 20 mg/kg dose (50.9 and 32.6% reduction in neurological score and infarct size, respectively). The use of such dose also significantly decreased neurological score and infarct volume at 72 h after tMCAO (Figures 4A,B). A significant improvement in neurological score was also observed at 24 h after MCAO with the 40 mg/kg dose (Figures 3A,B). Sham-operated animal group did not show any deficits. Qualitative assessment of TTC-stained sections of the 20 mg/kg dose group indicated a decrease of infarct size at both 24 and 72 h after MCAO (Figures 4A,B).

### Oxidative Stress Is Attenuated by RES-NPs Treatment

**FIGURE 5:**
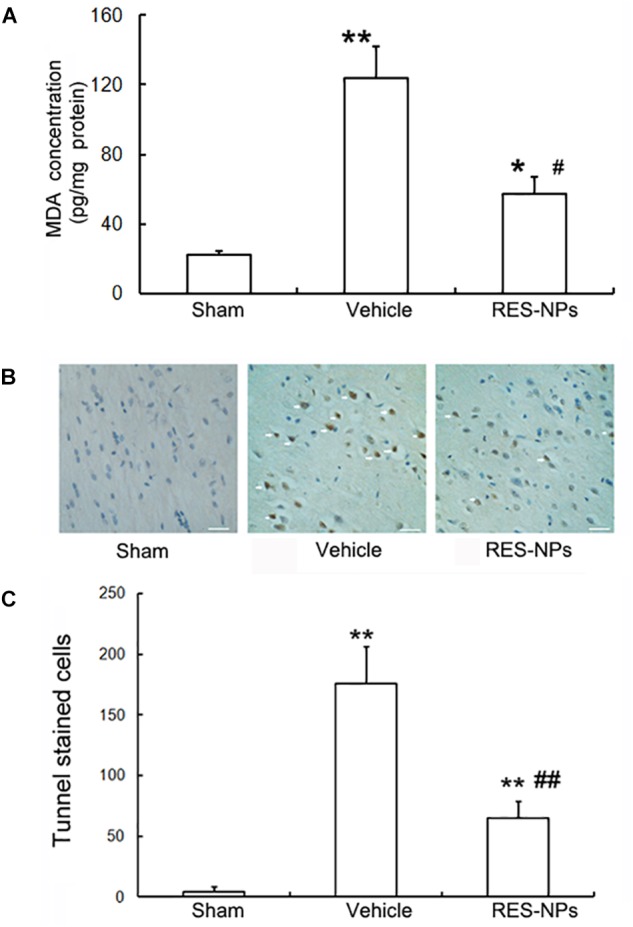
Inhibition of oxidative stress and apoptosis by RES-HSA-NPs (RES-NPs) in rats following ischemia-reperfusion. **(A)** RES-NPs attenuated oxidative stress in brain after I/R. Quantified (*n* = 4) MDA levels of the cerebral cortex from I/R side after 24 h reperfusion. The RES-NPs (20 mg/kg) treatment had significantly decreased cortex MDA levels compared with the vechicle (^∗∗^*P* < 0.01 vs. sham, ^##^*P* < 0.01 vs. vehicle, ANOVA). **(B)** Representative photomicrographs showing yellow-brown TUNEL staining cells (pointed by white arrows) in penumbral region of brain sections from Sham, vehicle and RES-NPs treated rats after 72 h reperfusion (*n* = 4). Scale bars = 100 μm. **(C)** Quantification of the TUNEL-staining cells. ^∗^*P* < 0.05 vs. sham, ^#^*P* < 0.05 vs. vehicle.

To evaluate the oxidative stress quantitatively, the MDA assay was applied to determine the level of lipid peroxidation products. The MDA levels in the cortical areas of the brains from tMCAO group significantly increased. Treatment with RES-HSA-NPs (20 mg/kg) significantly reduced MDA levels compared with vehicle-treatment (Figure 5A).

### RES-HSA-NPs Reduces Neuronal Apoptosis

Three days after reperfusion, neuronal apoptosis was investigated in peri-infarct zone by terminal deoxynucleotidyl transferase (TdT)-mediated dUTP-biotin nick end labeling (TUNEL) stain. Only a few TUNEL-positive cells (yellow brown) were observed in the sham group. I/R increased the number of TUNEL-positive cells, while 20 mg/kg dose of RES-HSA-NPs reduced the number of TUNEL-positive cells in peri-infarct zone (Figure 5B).

Quantitative analysis revealed that the number of TUNEL-positive cells in brain of 20 mg/kg RES-HSA-NPs treated rats was significantly fewer than in the vehicle treated group (68 vs. 169 apoptotic cells/0.1 mm^2^, *P* < 0.05) (Figure 5C).

## Discussion

Ischemic stroke is known as a top-killer for decades around the world. Oxidative stress, including excessive oxidation and insufficient elimination, has been proved to be a critical underlying molecular mechanism (Chamorro et al., 2016; Chamorro, 2018). Natural polyphenols including RES and curcumin, patent drugs such as edaravone injection, and endogenous substances like glutathione (GSH) and superoxide dismutase (SOD), have shown their enormous potential in prevention and treatment as effective antioxidants in experimental studies. However, none of these strategies was successful in clinical translation (Ghosh et al., 2014; Nagy and Nardai, 2017; Sun et al., 2018). Several reasons may explain the paradox. Firstly, both permanent ischemia and I/R models were applied in different experiments, which did not promise an effective blood flow to load the antioxidants into brain. Secondly, the half-life time of free antioxidants are short *in vivo*, which could result in variant concentrations in the brain. The hydrophobicity of some small molecular antioxidants and the poor penetration of antioxidative enzymes into brain also accounted for the unsuccessful application (Nagy and Nardai, 2017; Sun et al., 2018).

Recently, reperfusion therapy within a defined time-window has shown benefit in several types of IS. However, injury following the sudden recovery of brain blood flow will discount the benefit and make disastrous results (Sun et al., 2018). Therefore, it is urgent to explore protective treatment combined with reperfusion. Transient focal cerebral ischemia rat prepared with transient intraluminal filament MCAO was proved to be the ideal experimental model for ischemia and reperfusion (Lopez and Vemuganti, 2018).

Ischemia and reperfusion change the antioxidant status of brain tissue (Chamorro, 2018). Endogenous antioxidants levels in I/R brain tissue are rapidly reduced and kept below the baseline over a span of several days after blood reperfusion. Oxidative stress could breakdown the BBB and damage cellular macromolecules which triggered sequential neuron apoptosis (Chamorro et al., 2016; Chamorro, 2018). Exogenous supplementation of antioxidants is suggested to be an effective way in protecting the brain from I/R injury.

Previous studies have highlighted that RES plays an important role in attenuating several kinds of I/R injury including cerebral I/R injury (Liu et al., 2015). However, its short *in vivo* half-life and low tissue affinity hampered the practical use of free RES (Nabavi et al., 2014; Borges et al., 2018). Therefore, a repeated administration of RES before and post the procedure of I/R was applied in most studies. In most experiments, RES was usually administrated through oral or intraperitoneal routes with a considerable high dose (Nabavi et al., 2014; Liu et al., 2015).

Recent development in the technology of drug delivery provided a ray of hope to break the dilemma. It has been reported that, SOD modification with PEG or delivery by nanoparticles effectively treated I/R injury. Several kinds of carrier-based delivery of RES have been developed in recent years (Lu et al., 2009; Liu et al., 2015; Fan et al., 2018). Nanoparticles encapsulation based on chitosan, polymer micelles, liposome, and serum albumin improved the stability and *in vitro* biocompatibility of RES (Lu et al., 2009, 2013; Liu et al., 2015; Fan et al., 2018; Jhaveri et al., 2018; Trotta et al., 2018), which is beneficial for biomedical applications in protection against ultraviolet radiation, radiological injury, β-amyloid disease, and tumor (Lu et al., 2009; Yin et al., 2014; Doppalapudi and Mahira, 2017; Jhaveri et al., 2018). Moreover, the vitro antioxidative capacity of RES in nanoparticles is comparable to and better than that of free RES (Yin et al., 2014; Geng et al., 2017; Fan et al., 2018). In our previous publication, it was reported that RES encapsulated in polymeric micelles nanoparticles demonstrated better protection on rat cortical neuron culture than free RES (Lu et al., 2013). However, exogenous carriers such as chitosan, polymeric micelles, and liposomes demonstrated inevitable immunogenicity. HSA is an intrinsic protein and important biocarrier that transports many endogenous as well as exogenous substances (Jhaveri et al., 2018).

The concept of enhanced-penetration-and-retention (EPR) effect was first defined by Matsumura and Maeda (1986) to account for the accumulation of macromolecules in tumors. The drug delivery by kinds of carriers based on this strategy obtained great development and success especially in cancer therapy. BBB degeneration after brain ischemic reperfusion may lead to vasogenic edema (Sandoval and Witt, 2008). The phenomenon of increased permeability of the BBB conferred to regional accumulation of macromolecules, including albumin and immunoglobulins, during reperfusion after transient ischemia (He et al., 2018). Therefore, serum albumin, the intrinsic macromolecule without immunogenicity and toxicity, could be applied as the biocarrier to transport lipophilic antioxidative small molecule into I/R brain (Jhaveri et al., 2018).

The translation of animal results to humans relies on the similarity of the pathological processes between animal model and that of human patient. BBB degeneration after cerebral I/R was demonstrated as a biphasic or a continuous course in animal or in human studies, respectively (Merali et al., 2017). Recent studies with tighter experimental controls suggested a gradual increasing leakage of BBB during 24 h instead of continuous open for weeks (Veltkamp et al., 2005; Strbian et al., 2008). Small molecules and macromolecules also demonstrated different penetration across the BBB during I/R injury. Different methods of assessing BBB permeability and animal models also accounted for the variability of reported pattern of BBB leakage. In our study, a modified method with Albumin-Evans-Blue complex was used to confirm the regional accumulation of albumin in I/R brain within 24 h. This result provided the footstone for the application of RES-HSA-NPs in protection from cerebral I/R injury. The relatively acidic environment in I/R brain zone being considered, a PH dependent release pattern of RES from RES-HSA-NPs, revealed by *in vitro* cumulative release study, might optimize the *in vivo* behavior of RES-HSA-NPs. A single dose of 20 mg/kg RES-HSA-NPs via tail vein improved neurological outcomes and decreased infarct volume at 24 and 72 h in rats suffering transient middle cerebral occlusion. The underlying explanation would be that long-circulating and localized collection of RES-HSA-NPs attenuated oxidative stress and neuronal apoptosis after I/R (Chamorro, 2018; Yang J.-L. et al., 2018).

The efficacy of RES-HSA-NPs may due to prolonged circulation in blood, localization in I/R brain region, and sustained release pattern. This strategy could also be applied to other compounds which lack effective clinical translation in I/R situation.

## Conclusion

RES-HSA-NPs can improve neuronal outcomes after focal cerebral ischemia reperfusion by decreasing oxidative stress and neuronal apoptosis. RES-HSA-NPs may also provide benefits when used as an adjunctive treatment to reperfusion therapy. Future studies are warranted to evaluate the potential of this combination strategy in IS.

## Author Contributions

HX, YH, XLi, and XLu contributed to the conception and design of the study. HX, YH, WX, YC, YM, and XLu carried out the experiments. JZ and XLu prepared the manuscript.

## Conflict of Interest Statement

The authors declare that the research was conducted in the absence of any commercial or financial relationships that could be construed as a potential conflict of interest.
